# Digital infrastructure and intra-provincial balanced development: Evidence from China’s provincial panel data

**DOI:** 10.1371/journal.pone.0324132

**Published:** 2025-06-09

**Authors:** Shuping Lin, Lingling Zuo, Tao Cen

**Affiliations:** 1 College of International Economics and Trade, Ningbo University of Finance and Economics, Ningbo, China; 2 Ningbo Philosophy and Social Science Key Research Base “Research Base on Digital Economy Innovation and Linkage with Hub Free Trade Zones”, Ningbo, China; 3 Institute of Digital Economy and Industrial Innovation, Ningbo University of Finance and Economics, Ningbo, China; Gabriele d'Annunzio University of Chieti and Pescara: Universita degli Studi Gabriele d'Annunzio Chieti Pescara, ITALY

## Abstract

Unbalanced regional development poses a significant challenge to global economic growth. While existing studies have primarily concentrated on disparities between provinces, there is a notable lack of research addressing regional imbalances within a single province. In this paper, we use urban-level indicators to assess the level of intra-provincial regional balanced development in China. Based on provincial panel data from 2013 to 2022, we examine the impact of digital infrastructure on balanced regional development. We find that digital infrastructure significantly promotes balanced regional development using a fixed-effect regression model. This conclusion still holds after a series of robustness tests including instrumental variables, considering variable lag effects. Then we identify technological innovation and industrial structure upgrading as the primary mechanisms driving this relationship. Heterogeneity analysis reveals that the impact of digital infrastructure on balanced provincial development is more pronounced in regions with higher levels of urbanization, whereas the heterogeneity associated with financial development levels is statistically insignificant. Based on these findings, the paper offers policy recommendations aimed at leveraging digital infrastructure to further empower balanced development across provinces.

## Introduction

Unbalanced regional development is a common challenge faced by countries worldwide, particularly those with large jurisdictions [1,2]. Since China’s reform and opening-up in 1978, the country has made significant economic progress. By 2023, China’s GDP had reached approximately 18.3 trillion dollars, ranking second globally, with its per capita GDP at around 12,900 dollars—82 times higher than in 1978. However, the rapid economic growth has been accompanied by unbalanced regional development. In the early-stage of the reform and opening-up, China implemented an unbalanced development strategy, characterized by an economically advanced eastern region as the “center” and underdeveloped central and western regions as the “periphery”. This strategy has caused the economic gap between regions to persist and even widen [3]. Scholars have extensively explored the characteristics, influencing factors, and development pathways of China’s regional development imbalance, yielding a wealth of insights [3–6]. However, most studies have concentrated on inter-provincial development imbalances, while research on unbalanced regional development within provinces remains relatively underexplored. Intra-provincial unbalanced regional development is a typical phenomenon existed in many provinces. It is a crucial aspect of China’s overall regional development imbalance. For example, even as one of the most developed provinces in China, Guangdong Province experiences significant regional development imbalances. The gap in per capita GDP between the Pearl River Basin and non-Pearl River Basin regions continues to widen [7]. Therefore, addressing the intra-provincial unbalanced regional development has thus become an urgent issue that needs to be tackled.

As a product of the new generation of technological revolutions, digital technology fosters the transformation of economic production methods through its characteristics of sharing, inclusiveness, and extensive coverage. So, digital infrastructure has become a key engine of economic development. In recent years, China has implemented a series of major initiatives, including “Broadband China,” “New Infrastructure”, and “Digital China”. The development of digital infrastructure, represented by 4G/5G networks, fiber broadband, industrial internet, and data centers, has made substantial progress [8]. The development level of digital infrastructure in China continues to lead globally. According to the *Digital China Development Report (2023)*, by the end of 2023, China is expected to have 3.377 million 5G base stations and 23.02 million 10G PON ports with gigabit network service capabilities.

Moreover, while accelerating the development of internet infrastructure in the more developed eastern regions, China has also prioritized the construction and popularization of internet infrastructure in underdeveloped regions such as the central and western provinces, aiming to mitigate regional development imbalances [9]. Digital infrastructure, with its “super-temporal and super-spatial” characteristics that connect digital and geographic spaces, can effectively facilitate the flow of resources and promote balanced economic development [10]. Given its role as a critical strategic resource and growth engine, the question arises: Can digital infrastructure promote balanced regional development within provinces? Addressing this question is crucial for tackling the issue of unbalanced regional development in China.

Existing literature has explored the impact of digital infrastructure development on various aspects of economic growth, including economic development [11], technological innovation [12], employment [13], and carbon emissions [14]. Additionally, some studies have examined the effect of digital finance on regional balanced development [15,16]. However, there is a relative scarcity of research focusing on the influence of digital infrastructure on regional balance, particularly within provinces. Moreover, the mechanisms through which digital infrastructure affects intra-provincial balanced development require further investigation. To address this gap, this paper uses panel data from 31 provinces in China spanning from 2013 to 2022. Employing a two-way fixed-effects model, the study examines the relationship between digital infrastructure development and regional balance within provinces.

The main contributions of this paper are as follows. First, it evaluates the impact of digital infrastructure construction on intra-provincial balanced development. It finds that digital infrastructure significantly promotes provincial balanced regional development, thereby enriching the theoretical research on both digital infrastructure and balanced regional development. Second, it identifies the mechanisms through which digital infrastructure affects provincial balanced development, specifically through technological innovation and industrial structure upgrading. This enhances the understanding of how digital infrastructure contributes to regional balance. Third, the paper analyzes the heterogeneity of the impact of digital infrastructure on provincial balanced development. The results indicate that greater financial support and higher levels of transportation convenience amplify the effect of digital infrastructure construction on provincial balanced development, broadening the research perspective on its regional implications.

## Literature review and theoretical framework

### Literature review

#### The economic effects of digital infrastructure.

Existing literature primarily explores the impact of digital infrastructure on various aspects such as economic development, technological innovation, industrial structure upgrading, social welfare, and environmental protection. In terms of economic growth, digital infrastructure offers significant advantages, including broad penetration and extensive coverage. It fosters high-quality economic development through mechanisms such as industrial structure optimization, market integration, and enhanced production efficiency [17]. Datta and Agarwal (2004) dynamically examined the impact of telecommunications investment on economic productivity using data from 22 OECD countries, confirming the positive correlation between telecommunications infrastructure and economic growth [18]. Hussain et al. (2023) utilized data from 110 developing countries, demonstrating that digital infrastructure (ICT) plays a crucial role in the sustainable development of economies [19]. Regarding technological innovation, digital infrastructure facilitates the dissemination of innovation factors across regions and industries by efficiently allocating innovation resources and improving knowledge flow, thereby enhancing technological innovation efficiency [20]. Osei (2024) studied the relationship between digital infrastructure and innovation in 28 African countries, finding that digital infrastructure promotes technological innovation through human capital accumulation [21]. Tian and Lu (2023) found that digital infrastructure significantly encourages cross-regional collaborative innovation within enterprises. Concerning industrial structure upgrading, digital infrastructure can directly or indirectly promote industrial upgrading by improving total factor productivity and technical efficiency [22]. Mitra et al. (2016) found that digital infrastructure has a notable impact on the production efficiency of manufacturing, particularly in highly competitive industries [23]. Wu et al. (2023) showed that digital infrastructure has effectively facilitated the upgrading of industrial structures in Chinese cities, with a more pronounced effect in larger cities [24]. On the social welfare front, digital infrastructure construction significantly impacts employment and helps narrow the income gap. Ndubuisi et al. (2021) found that digital infrastructure construction has a notable positive effect on employment in the service sector [13]. Jiang and Jin (2024) concluded that digital infrastructure can significantly reduce the urban-rural income gap and has a spatial spillover effect [25]. Regarding environmental impact, the effects of digital infrastructure are mixed. On one hand, digital infrastructure construction leads to increased energy consumption and higher carbon emissions [26]. On the other hand, it promotes the upgrading and transformation of traditional industries, which can improve carbon performance [27].

#### Factors affecting balanced regional development.

There is an extensive body of research addressing the factors contributing to regional economic imbalances, with studies examining the issue from various perspectives, including economic geography, government intervention, technological progress, industrial structure, and factor mismatch. From an economic geography perspective, regions differ in terms of resources and location conditions, which often leads to the concentration of industries in areas with better factors, thereby exacerbating regional economic imbalances [28]. Gallup et al. (1999) argued that geographical location and climate significantly influence the uneven development of regional economies [29]. Liu and Wang (2009) highlighted the role of natural geographical factors, transportation infrastructure, and locational advantages in shaping China’s regional development disparities [30]. In terms of government intervention, Yang (2002) focused on the imbalance between urban and rural areas, identifying government policies as the primary driver of unequal development [31]. Wei and Liu (2004) examined the evolution of regional development gaps in China, concluding that these imbalances are closely linked to regional development strategies and national policies [32]. Regarding technological progress, Belton Fleisher et al. (2017) explored the role of human capital in economic growth, finding that human capital, as an input factor, contributes to regional disparities by boosting labor productivity and output levels [33]. Tang and Chen (2020) argued that the model of unbalanced development resulting from technological introduction has played a key role in China’s rapid economic growth [34]. They observed that during technological transitions, regional gaps initially narrow before widening again. In terms of industrial structure, Azapagic (2004) developed a linkage model of regional, inter-industry, and cross-regional industrial structures, concluding that reducing regional economic imbalances is closely related to the rationalization of industrial division of labor [35]. Pei (2022) emphasized the significance of both the rationalization and sophistication of industrial structures in addressing regional economic gaps, noting that the impact of industrial rationalization on reducing disparities has grown over time [36]. Finally, factor mismatch is another key contributor to regional imbalances. Krugman and Venables (1995) pointed out that the misallocation of resources due to mismatches between factors and industries, as well as between factors and locations, is a major cause of regional economic disparities [37]. Fan (2004) noted that China’s relatively low level of marketization has led to the concentration of manufacturing in coastal areas, preventing its transfer to central and western regions, thereby deepening regional disparities [38].

### Theoretical framework

#### Digital infrastructure and balanced regional development.

Digital infrastructure fosters balanced regional development by influencing the accumulation and allocation of both physical and labor capital among cities within a province. On the one hand, information infrastructure, which is centered around information and communication technology, is characterized by its wide-reaching penetration. Traditional industries leverage this infrastructure to enhance technological innovation, upgrade industrial structures, and expand production capacities through its “penetration effect.” This, in turn, improves total factor productivity [39], which is beneficial for capital accumulation in cities. Notably, cities with relatively underdeveloped information infrastructure experience greater marginal benefits from its construction. As such, the development of information infrastructure can effectively reduce the performance gap between urban agglomerations. On the other hand, digital infrastructure, with its “super-time and super-space” characteristics that link digital and geographic spaces, acts as a “digital bridge” between regions. This facilitates the free flow of factors across regions, thereby improving the overall allocation of resources [40]. Additionally, the construction of digital infrastructure accelerates the dissemination and exchange of technology and knowledge between regions, promoting faster and more efficient diffusion of technological innovations. This enhances inter-city connectivity and cooperation, ultimately contributing to more balanced regional development [41]. Based on the above analysis, we propose the following hypotheses:

***Hypothesis 1:*** *Digital infrastructure promotes intra-provincial balanced regional development.*

#### Mechanisms of digital infrastructure affecting balanced regional development.

First, digital infrastructure construction impacts the balanced development of provinces by fostering technological innovation. Digital infrastructure supports the efficient and stable operation of various digital applications, providing a solid foundation for technological advancements. The interconnected and shared nature of digital infrastructure enables the efficient and instant flow of data, overcoming the time and space barriers that typically hinder information exchange. This enhances the efficiency of knowledge transfer, accelerating the pace of technological innovation [20]. Additionally, digital infrastructure connects data, information, and knowledge resources across regions, allowing innovators to access a broader range of resources and reduce the costs associated with technological development [42]. Technological innovation serves as the most critical driver of regional economic growth, exhibiting strong positive externalities and generating spillover effects. Digital infrastructure, in particular, facilitates the diffusion of technology from advanced regions to less developed areas [43], achieving interregional technological convergence that effectively narrows economic disparities across regions [44,45]. On the one hand, technologically lagging regions can accelerate their innovation capabilities through imitation and technology transfer from advanced regions, realizing rapid technological upgrading and economic convergence [46]. On the other hand, over the long term, sustained growth driven by technological breakthroughs in innovation-intensive regions is likely to encounter diseconomies of scale due to congestion and rising factor costs. This may prompt the diffusion of developmental resources to neighboring areas, thereby alleviating interregional development disparities [47]. Additionally, technological innovation effectively stimulates the vitality of emerging industries and new business models. It accelerates the integration of market factors and enhances resource allocation efficiency [48], contributing to balanced regional development.

Second, the construction of digital infrastructure contributes to the balanced development of provinces by promoting the upgrading of industrial structures. Digital infrastructure has fundamentally altered economic development models, acting as a critical force for industrial upgrading. This upgrading involves both the rationalization and transformation of industrial structures. In terms of rationalizing industrial structures, digital infrastructure facilitates the free flow of factors across regions, driving the optimal allocation of labor, capital, and technology [49]. This optimization creates favorable conditions for the growth of high-tech and knowledge-based industries, thus promoting the rationalization of industrial structures [50]. Regarding industrial upgrading, digital infrastructure enables the widespread adoption of digital technologies across industries, transforming and enhancing traditional sectors, improving their innovation capacity and efficiency, and fostering regional industrial upgrading [51]. Additionally, the digital economy itself is a high-tech industry, and the expansion of digital infrastructure significantly boosts the scale of this sector, increasing the proportion of high-tech industries within regional economies. The process of industrial upgrading involves both a “transfer effect” and a “catch-up effect.” As industries in developed regions transfer some of their operations to less developed areas, the latter experience accelerated economic growth, known as the “transfer effect” [52]. Meanwhile, backward regions can adopt industrial technologies from developed regions at lower costs, enabling them to achieve industrial upgrading more affordably, a phenomenon referred to as the “catch-up effect” [53]. Both effects contribute to narrowing the regional development gap, as the flow of technology and industry from advanced regions to underdeveloped areas helps promote more balanced economic growth. Meanwhile, the industrial structure upgrading can stimulate industrial innovation and facilitate the cross-sectoral flow of production factors such as technology and knowledge among industries, thereby fostering a new development paradigm characterized by regional division of labor collaboration and complementary advantages. This process ultimately enhances the overall efficiency of regional economies. Particularly in less-developed regions where industrial structures remain relatively backward, industrial restructuring enables these areas to capture greater growth dividends through structural transformation, thus achieving regional coordinated development [54–56].

Therefore, we propose

***Hypothesis 2:*** *Digital infrastructure promotes intra-provincial balanced regional development through technological innovation and industrial structure upgrading.*

## Research design

### Econometric model

In order to examine the impact of digital infrastructure on intra-provincial balanced regional development, we establish the benchmark regression model:


$${lnUNB}_{it}=\alpha_{0}+\alpha_{1}*{lnDIF}_{it}+\alpha_{2}*X_{it}+\mu_{i}+\theta_{t}+\varepsilon_{it}$$ (1)

Where ${UNB}_{it}$ represents the level of unbalanced regional development of province i in year t. ${DIF}_{it}$ represents the level of development of digital infrastructure, $X_{it}$ represents a series of control variables. $\mu_{i}$ and $\theta_{t}$ represent the individual fixed effect and time fixed effect, respectively. $\varepsilon_{it}$ is a random interference term.

In order to verify the mechanism of digital infrastructure in promoting balanced provincial development, we use the following econometric model:


$${lnM}_{it}=\beta_{0}+\beta_{1}*{lnDIF}_{it}+\beta_{2}*X_{it}+\mu_{i}+\theta_{t}+\varepsilon_{it}$$ (2)

where $M_{it}$ is a mechanism variable, including technological innovation and industrial structure upgrading.

### Variable construction

#### Explained variable.

The explained variable is the level of intra-provincial unbalanced regional development (UNB). Following Cowell (2000) [57], the Theil index is used to measure intra-provincial regional imbalance. The formula for the Theil index is:


$$\text{THEIL}=\sum_{j=1}^{n}y_{j}log\frac{y_{j}}{p_{j}}$$ (3)

where n is the total number of cities in the province; $y_{j}$ denotes the proportion of city j’s GDP relative to the province’s total GDP; $p_{j}$ is the proportion of city j’s population relative to the total population of the province. The higher the THEIL index, the more unbalanced regional development.

The formula for calculating regional income gap is

For robustness checks, we use the regional income gap measure from Akita and Miyata (2000) in the regression analysis [58]. The formula for calculating regional income gap is:


$$\text{CV}=\frac{1}{\overset{―}{g}}\sqrt{\sum_{i=1}^{n}{\left(g_{i}-\overset{―}{g}\right)}^{2}/(n-1)}$$ (4)

where $g_{j}$ represents the GDP per capita of city j in the province, and $\overset{―}{g}$ is the average GDP per capita across all cities in the province. Note that the calculation of the Theil index and the regional income gap for the four municipalities—Beijing, Shanghai, Tianjin, and Chongqing—is based on district (county) data for each municipality.

#### Explanatory variable.

The explanatory variable is the level of digital infrastructure development (DIF). Existing literature employs two approaches to measure DIF: the single indicator method and the comprehensive indicator system method. The single indicator method uses one measure, such as per capita long-distance optical cable length or telecommunications fixed asset investment, while the comprehensive system constructs a development index based on multiple indicators. Given the limitations of a single indicator, this paper adopts the comprehensive indicator system, which includes five indicators, such as mobile phone base station density (Table 1), and applies the entropy method to calculate the digital infrastructure development index.

**Table 1:** Indicators of evaluating digital infrastructure development level.

Indicator	Unit	Direction	Weight
Mobile phone base station density	pcs per 10,000 people	+	0.233
Optical cable density	km per 10,000 people	+	0.262
Mobile phone penetration rate	units per 100 people	+	0.151
Internet broadband penetration rate	households per person	+	0.175
Internet port density	pcs per person	+	0.179

#### Control variables.

Building on the research of Yu et al. (2022) [59], four control variables are introduced: economic development level (PGDP), marketization level (ML), transportation convenience (FTD), and per capita education expenditure (PEE). PGDP is represented by GDP per capita, ML is captured using the marketization index, FTD is measured by per capita road area, and PEE is defined as per capita education expenditure.

#### Mechanism variables.

The mechanism variables include technological innovation and urbanization. Technological innovation (INV) is measured by the number of patents granted per 10,000 people; urbanization (UBR) is measured by the proportion of urban residents. The industrial structure upgrading level (ISU) refers to the research of Chang et al. (2019) and constructs an industrial structure upgrading index to measure the level of industrial structure upgrading [60]. The calculation method is as follows:


$$ISU=\sum_{\tau =1}^{3}q_{\tau }\times i=q_{1}*1+q_{2}\times 2+q_{3}\times 3$$ (4)

In the above formula, $q_{\tau }$ represents the output share of the τ-th industry. A higher ISU index indicates a more advanced and developed industrial structure.

### Data sources and descriptive statistics

Most of the data are sourced from the *China Statistical Yearbook* and the statistical yearbooks of various provinces. The marketization index data for 2013–2019 are obtained from the *China Provincial Marketization Index Report* (2021) [61]. The missing marketization index values for 2020–2022 are estimated using the average growth rate. To mitigate potential heteroscedasticity and nonlinearity arising from differences in the measurement units of the variables, all variables are log-transformed. The descriptive statistics for each variable are presented in Table 2.

**Table 2:** Descriptive statistics.

Variable	Obs	Mean	Std	Min	Max
lnTHEIL	310	-3.29	0.62	-5.26	-1.89
lnCV	310	-0.79	0.36	-1.73	0.10
lnDIF	310	-5.91	0.66	-8.04	-4.82
lnPGDP	310	10.96	0.44	9.11	12.16
lnML	310	2.03	0.52	-4.61	2.55
lnFTD	310	1.74	0.39	0.53	2.62
lnPEE	310	-1.49	0.36	-2.17	-0.14
lnINV	310	2.19	1.06	-0.95	4.53
lnISU	310	0.87	0.05	0.78	1.04
lnUBR	310	4.07	0.22	3.17	4.50

## Empirical result

### Benchmark regression result

Table 3 presents the empirical findings from the baseline regression analysis. Column (1), which excludes control variables, reveals that the coefficient for the digital infrastructure development index is negatively significant at the 1% level. This suggests that enhanced digital infrastructure may contribute to reducing the uneven development across provinces. Columns (2) through (5) incrementally incorporate four control variables: economic development level, marketization level, transportation convenience level, and per capita education expenditure level. Throughout these columns, the coefficient for the digital infrastructure development index consistently remains negative and significant at the 1% level, demonstrating the robustness of the benchmark empirical results. This finding is economically significant as well. For instance, the coefficient in column (5) is -0.207, suggesting that a 1% increase in digital infrastructure level is associated with a 0.207% decrease in the regional development imbalance index. This decrease accounts for 6% of the mean of the explained variable, underscoring the substantial impact of digital infrastructure on regional development disparities. In other words, digital infrastructure construction can promote balanced regional development. Hypothesis 1 holds.

**Table 3:** Benchmark regression results.

Variable	(1)	(2)	(3)	(4)	(5)
	lnTHEIL	lnTHEIL	lnTHEIL	lnTHEIL	lnTHEIL
lnDIF	-0.288^***^ (0.067)	-0.287^***^ (0.067)	-0.284^***^ (0.067)	-0.229^***^ (0.071)	-0.207^***^ (0.071)
lnPGDP		-0.086 (0.068)	-0.070 (0.068)	-0.054 (0.068)	0.019 (0.074)
lnML			-0.080^**^ (0.038)	-0.065^*^ (0.039)	-0.034 (0.040)
lnFTD				-0.254^**^ (0.120)	-0.273^**^ (0.119)
lnPEE					-0.491^**^ (0.206)
Constant	-5.176^***^ (0.475)	-4.250^***^ (0.878)	-4.258^***^ (0.873)	-3.690^***^ (0.908)	-5.220^***^ (1.105)
Province FE	Yes	Yes	Yes	Yes	Yes
Year FE	Yes	Yes	Yes	Yes	Yes
Obs	310	310	310	310	310
R^2^	0.38	0.39	0.40	0.41	0.42

Note:

*** ,

** , and

* denote significance at the 1%, 5%, and 10% levels, respectively, with standard errors in parentheses. The same applies to the following text.

### Endogeneity test

Considering the potential endogeneity issues in the benchmark regression model, including reverse causality and omitted variables. This study employs two methods to address these concerns.

#### Instrumental variable (IV).

Drawing on the approach by Zhao et al. (2020) [62], this paper constructs an IV using interaction term between the number of post offices per province in 1998 and the nationwide number of Internet users in the previous year. This term serves as an instrumental variable for the digital infrastructure of each province in the respective year. The results, presented in column (1) of Table 4, show that both the Anderson LM and the Cragg-Donald Wald F statistics successfully reject the null hypotheses of unidentifiability and weak instrumental variables, confirming the appropriateness of the selected instrumental variable. The IV regression coefficients are significant at the 1% level, reinforcing the conclusion that the development of digital infrastructure contributes to the balanced development of provinces.

**Table 4:** Endogeneity tests.

Variable	(1)	(2)
	lnTHEIL	lnTHEIL
L.lnDIF		-0.284^***^ (0.0751)
lnDIF	-0.302^***^ (0.1104)	
Control	Yes	Yes
Province FE	Yes	Yes
Year FE	Yes	Yes
Obs	310	279
LM statistic	70.20 [0.0000]	
Wald F statistic	92.121 {16.38}	

Note: The values in brackets represent p-values, while the values in curly braces indicate the critical values at the 10% level of the Stock-Yogo weak identification test.

#### Considering the lag-term.

Following the research of Yin et al. (2023) [63], this study lags the core explanatory variables and control variables by one period in the benchmark regression model to mitigate the effects of endogeneity. The regression results, detailed in column (2) of Table 4, reveal that the coefficient for digital infrastructure remains negative and is statistically significant at the 1% level. This consistency with the benchmark regression results underscores the reliability of the research conclusions.

### Robustness test

We employ a series of robustness tests to validate the findings: Firstly, we replace the Theil index with the regional income gap (calculated as per Formula 4) as the dependent variable for regression, with results shown in column (1) of Table 5. Secondly, we substitute the core explanatory variable, digital infrastructure development level, with Internet penetration rate, documenting outcomes in column (2) of Table 5. Thirdly, to mitigate the influence of outliers, we trim and truncate the top and bottom 2.5% of Theil index samples, presenting the adjusted results in columns (3) and (4) of Table 5. Fourthly, we apply a random effects model to regress model (1), with findings provided in column (5) of Table 5. Lastly, we employ more rigorous province-clustered robust standard errors for the regression, and the results are reported in column (6) of Table 5.

**Table 5:** Robustness tests.

Variable	(1)	(2)	(3)	(4)	(5)	(6)
	lnCV	lnTHEIL	lnTHEIL	lnTHEIL	lnTHEIL	lnTHEIL
lnDIF	-0.102*** (0.0394)	-0.477*** (0.0934)	-0.215*** (0.0715)	-0.217*** (0.0780)	-0.234*** (0.0721)	-0.207* (0.1059)
Control	Yes	Yes	Yes	Yes	Yes	Yes
Province FE	Yes	Yes	Yes	Yes	Yes	Yes
Year FE	Yes	Yes	Yes	Yes	Yes	Yes

All the robustness checks consistently support Hypothesis 1, reinforcing the reliability of our conclusions.

### Mechanism analysis

Hypothesis 2 posits that digital infrastructure development promotes balanced provincial development primarily through technological innovation and industrial structure upgrading. The empirical results supporting this hypothesis are detailed in Table 6. Column (1) displays the regression results for the impact of digital infrastructure development on technological innovation, indicating a significant promotion of technological innovation by digital infrastructure. Regions with advanced technology levels have facilitated the development of neighboring areas through a radiative effect of technological innovation, thereby reducing the regional economic disparities. Column (2) shows the regression results for the impact of digital infrastructure on industrial structure upgrading, with its coefficient significant at the 1% level. This suggests that digital infrastructure significantly fosters the upgrading of industrial structure, which in turn promotes regional balanced development through “transfer effects” and “catch-up effects.” Thus, Hypothesis 2 is substantiated.

**Table 6:** Results of mechanism analysis.

Variable	(1)	(2)
	lnINV	lnISU
lnDIF	0.353*** (0.0691)	0.011*** (0.0043)
Control	Yes	Yes
Province FE	Yes	Yes
Year FE	Yes	Yes

### Heterogeneity analysis

The construction of digital infrastructure heavily depends on substantial financial support. According to Li (2022) [64], more developed financial markets can offer greater medium- and long-term loans, thereby accelerating the construction of digital infrastructure. Moreover, urbanization significantly contributes to this development. Hu et al. (2021) note that urbanization increases the demand for digital technologies, which in turn drives the construction of digital infrastructure [65]. Additionally, urbanization provides essential support such as technology, capital, and talent for digital infrastructure projects [66]. Consequently, higher levels of financial market development and urbanization are beneficial for digital infrastructure development, which promotes balanced provincial development.

We divide our samples into two groups according to the median level of financial development and urbanization development, and then conducts group regression. The financial development level is measured by the ratio of the loan balance of financial institutions to the GDP of the year (FS). The regression results are shown in Table 7. Columns (1) and (2) are the regression results of the financial development level grouping. It can be seen that the development of digital infrastructure in regions with high financial development levels has a greater impact on provincial balanced development, and the coefficient is more significant. However, the seemingly unrelated regression (SUR) and Fisher’s combination tests indicate no significant differences in regression coefficients between the two groups. This is likely due to the rapid development of China’s digital inclusive finance in recent years, which has lowered the threshold for financial services, expanded their coverage, and enhanced their accessibility, thereby providing more diversified financial support for the construction of digital infrastructure in less developed regions [67]. Columns (3) and (4) are the regression results of urbanization development level. It is evident that digital infrastructure development in highly urbanized regions significantly promotes provincial balanced development, whereas its impact in less urbanized regions is not significant, and the seemingly unrelated regression and Fisher’s combination tests also indicate significant differences between the two. Therefore, the impact of digital infrastructure development on provincial balanced development exhibits heterogeneity in urbanization levels, meaning that the higher the level of urbanization, the more significant the effect of digital infrastructure development on provincial balanced development, while the heterogeneity in financial development levels is not significant.

**Table 7:** Results of heterogeneity analysis.

Variable	(1)	(2)	(3)	(4)
	High FS	Low FS	High UBR	Low UBR
	lnTHEIL	lnTHEIL	lnTHEIL	lnTHEIL
lnDIF	-0.298** (0.1320)	-0.147* (0.0821)	-0.529*** (0.1142)	0.160 (0.1211)
Control	Yes	Yes	Yes	Yes
Province FE	Yes	Yes	Yes	Yes
Year FE	Yes	Yes	Yes	Yes
SUR Test	0.27		3.17*	
Fisher’s Combination Test	-0.151		-0.688***	

## Conclusion and policy implications

This paper addresses the critical issue of regional unbalanced development in China, focusing specifically on provincial disparities. It leverages provincial panel data from 2013 to 2022 to explore how digital infrastructure development influences provincial balance. The analysis underscores the burgeoning nature of digital infrastructure construction in China and examines its effects through various analytical lenses, including the use of instrumental variables, lagged models, and robustness tests. The findings reveal that digital infrastructure development significantly mitigates provincial imbalances. Furthermore, the study identifies two primary mechanisms through which digital infrastructure impacts provincial balance: technological innovation and industrial structure upgrading. Additionally, the research highlights that the higher the level of urbanization, the more significant the effect of digital infrastructure development on provincial balanced development, while the heterogeneity in financial development level is not significant. Based on the above research conclusions, the following policy recommendations are put forward.

First, strengthening digital Infrastructure construction. Digital infrastructure serves as a pivotal engine for economic growth and a crucial lever for regional balance. The government should bolster investments in digital infrastructure, clearly define construction objectives and key tasks at each phase, and devise practical, medium- to long-term plans. These plans should facilitate active participation from private capital in both the construction and operation phases of digital infrastructure projects. Special attention should be given to central and western regions to diminish disparities with the more developed eastern areas, thus fostering nationwide coordinated development.

Second, leveraging the spillover effects of digital infrastructure. It is vital to maximize the benefits of digital infrastructure through enhanced information and technology sharing. The government should enact policies that boost the role of digital infrastructure in regional innovation, fostering technological spillovers and coordinated economic development. Further integration of digital technology with traditional industries is essential to accelerate industrial transformation and close regional development gaps. There should also be efforts to empower urban industrial development with digital technologies, fostering the growth of new industries, formats, and models driven by data processes. Moreover, developing digital infrastructure that serves both urban and rural areas will encourage the cross-regional integration of data resources, supporting the unified development of these areas.

Third, building a multi-level financial service system and advancing urbanization. The government should enhance the coordination of financial and fiscal policies, encouraging various financial institutions to provide credit support for new information infrastructure projects. In particular, greater efforts should be made to develop inclusive finance to better serve the construction of digital infrastructure in less developed regions. Additionally, advancing the new urbanization strategy will gradually elevate urbanization levels, providing necessary resources to support digital infrastructure projects. This approach will not only strengthen emerging urban digital economies but also stimulate digital infrastructure development from the demand side through expanded and strengthened new industries.
